# Estimating feedforward vs. feedback control of speech production through kinematic analyses of unperturbed articulatory movements

**DOI:** 10.3389/fnhum.2014.00911

**Published:** 2014-11-11

**Authors:** Kwang S. Kim, Ludo Max

**Affiliations:** ^1^Speech and Hearing Sciences, University of WashingtonSeattle, WA, USA; ^2^Haskins LaboratoriesNew Haven, CT, USA

**Keywords:** speech production, articulation, sensorimotor control, feedback, kinematics

## Abstract

To estimate the contributions of feedforward vs. feedback control systems in speech articulation, we analyzed the correspondence between initial and final kinematics in unperturbed tongue and jaw movements for consonant-vowel (CV) and vowel-consonant (VC) syllables. If movement extents and endpoints are highly predictable from early kinematic information, then the movements were most likely completed without substantial online corrections (feedforward control); if the correspondence between early kinematics and final amplitude or position is low, online adjustments may have altered the planned trajectory (feedback control) (Messier and Kalaska, 1999). Five adult speakers produced CV and VC syllables with high, mid, or low vowels while movements of the tongue and jaw were tracked electromagnetically. The correspondence between the kinematic parameters peak acceleration or peak velocity and movement extent as well as between the articulators' spatial coordinates at those kinematic landmarks and movement endpoint was examined both for movements across different target distances (i.e., across vowel height) and within target distances (i.e., within vowel height). Taken together, results suggest that jaw and tongue movements for these CV and VC syllables are mostly under feedforward control but with feedback-based contributions. One type of feedback-driven compensatory adjustment appears to regulate movement duration based on variation in peak acceleration. Results from a statistical model based on multiple regression are presented to illustrate how the relative strength of these feedback contributions can be estimated.

## Introduction

Current models of sensorimotor control suggest that movements are executed through a combination of feedforward and feedback control mechanisms (Kawato and Wolpert, 1998; Desmurget and Grafton, 2000; Wolpert and Flanagan, 2001, 2010). One approach to investigating the relative contributions of these separate mechanisms has involved perturbing various types of goal-directed movements and measuring any compensatory responses (Folkins and Abbs, 1975, 1976; Abbs and Gracco, 1984; Gracco and Abbs, 1985; Tremblay et al., 2003; Feng et al., 2011). An alternative approach—that may be ecologically more valid for movements that typically do not encounter external perturbations—involves estimating the feedforward and feedback components from natural, unobstructed movements. For example, unperturbed limb or eye movements have been analyzed with statistical methods focusing on the relationship between a movement's early kinematics and its endpoint (Messier and Kalaska, 1999; Heath et al., 2004; West et al., 2009). Specifically, these studies determined the correspondence between early movement kinematics (peak acceleration, peak velocity, and spatial coordinates at those landmarks) and final movement kinematics (total movement amplitude and spatial coordinates at movement endpoint). If final movement amplitudes and endpoints are highly predictable from the early kinematic information, it is likely that, once initiated, the movements were completed without substantial online corrections. That is, the movements were largely preplanned and executed in a feedforward manner. If, on the other hand, the correspondence between early kinematics and final amplitude or position is low, online adjustments may have altered the originally planned movement characteristics. In each of the aforementioned studies, results showed high degrees of correspondence between the early and final kinematics, thus suggesting that the parameters of limb and eye movements had already been determined at movement onset, and that such movements are driven primarily by feedforward mechanisms (Messier and Kalaska, 1999; Heath et al., 2004; West et al., 2009).

With regard to the sensorimotor control of *speech production*, the extent to which movements are preplanned (feedforward) or corrected online (feedback) remains poorly understood and contentious. Yet, quantitative methods that could be used to determine and compare the weighting of feedforward vs. feedback strategies across different speaker groups (e.g., individuals with sensorimotor speech disorders) and different speaking situations (e.g., varying in automaticity, communicative environment, or feedback conditions) would be of great scientific and clinical value. Stuttering, as one example, is a speech disorder that has been hypothesized to be directly related to deficiencies in sensorimotor integration that lead to imbalances in the affected individuals' reliance on feedforward vs. feedback strategies (Max, 2004; Max et al., 2004). Although some older studies on typical speech motor control have documented that there is a strong positive relationship between jaw or tongue movement peak velocity and overall amplitude, those results were presented and discussed in the context of estimating the effectors' mass-normalized stiffness or general variations in velocity for different phonetic contexts (Kuehn and Moll, 1976; Ostry et al., 1983; Munhall et al., 1985; Ostry and Munhall, 1985).

Here, we examined the feasibility of applying to speech movements a set of procedures and analyses previously used to estimate the weighting of feedforward vs. feedback control in studies of limb and eye movements. We used electromagnetic midsagittal articulography (EMA) to transduce tongue and jaw movements during sentence-level speech. Directly based on the work by Messier and Kalaska (1999), we measured kinematic landmarks (peak acceleration, peak velocity, movement endpoint) and the effectors' two-dimensional spatial coordinates at the time of those landmarks. To test the correspondence between early kinematics and final movement characteristics, we computed bivariate correlation coefficients (and the corresponding coefficients of determination) for the relationship between early kinematic variables (the magnitude of peak acceleration and peak velocity, and the spatial coordinates at the time of peak acceleration and peak velocity) and endpoint variables (movement amplitude and the spatial coordinates at movement endpoint). In addition, a statistical technique based on multiple regression was used as a separate approach to examine the possibility of directly quantifying the contribution of feedback-based mechanisms. If an additional variable (e.g., movement duration in Messier and Kalaska, 1999) correlates positively with peak acceleration or velocity across movements to different targets but negatively across movements to the same target, and if adding this variable to the regression model that predicts endpoint characteristics from initial kinematics results in a statistically significantly greater coefficient of determination, then this added variable may reflect feedback-based compensatory adjustments. That is, online adjustments in this variable may have been implemented during movement execution to compensate for variability in the achieved parameters of peak acceleration or peak velocity.

## Methods

### Subjects

The subjects were five healthy adult men with no diagnosed communication or other disorders and with normal hearing (pure tone behavioral thresholds at or below 20 dB HL for the octave frequencies 250–8000 Hz). Subjects ranged in age from 18 to 48 years (*M* = 30.8, *SD* = 12.07), and all were native speakers of American English.

### Procedure

All subjects gave informed consent prior to participating, and all procedures were approved by the Institutional Review Boards of the University of Connecticut (where the data were collected) and the University of Washington (where the data were analyzed).

Each subject produced 10–15 trials (depending on the holding time of the adhesive used to attach motion tracking sensors to the tongue—see below) of a block of 36 different phrases with embedded consonant-vowel (CV) and vowel-consonant (VC) target syllables. The rationale for including, and comparing, CV and VC syllables was based on the hypothesis that the underlying control mechanisms may differ between articulatory closing movements for VC syllables (e.g., the tongue tip moving up and coming to a stop against a mechanical obstruction such as the alveolar ridge in a production of “*at*”) and articulatory opening movements for CV syllables (e.g., the tongue tip moving down to position that is not mechanically constrained in a production of “*tea*”) (Kuehn and Moll, 1976; Löfqvist and Gracco, 2002; Perrier et al., 2003; Fuchs et al., 2006).

Target syllables were constructed by combining two voiceless stop consonants (/t, k/) and one voiceless fricative (/s/) with high, mid, and low front (/i, ε, æ/) or back (/u, ɔ, ɑ/) vowels in an attempt to obtain distinctly different movement distances similar to the limb movement paradigm used by Messier and Kalaska (1999). Thus, there were 36 different target syllables (2 syllable types × 3 consonants × 6 vowels), and each target syllable was embedded in one of 6 possible carrier phrases (for each combination of syllable type and consonant there was a specific carrier phrase, and then all 6 vowels were used in that particular syllable structure in that particular carrier phrase).

The phrases were constructed such that (a) the consonants immediately preceding and following the target vowel always shared the same place and manner of articulation, and (b) the vowel preceding the consonant of a *CV* target or following the consonant of a *VC* target was always schwa. These sentence construction constraints served to facilitate movement segmentation in the analysis stage by ensuring clear reversals in effector movement direction before and after the opening (for CV syllables) or closing (for VC syllables) movements of interest. The following carrier phrases were included:

**Table:** 

He said a t*V* to me.	He said *V*t again.
He spoke a k*V* quietly.	He spoke *V*k again.
He says a s*V* so well.	He says *V*s again.

To avoid confusion due to ambiguous orthographic spelling of the CV and VC syllables (e.g., representing /æ/ vs. /ɑ/ and /ɔ/ vs. /ɑ/), subjects were first taught the phonetic symbols for the 6 target vowels by means of sample words. During data recording, the phrase for each trial appeared on a computer monitor with the target vowel represented in the corresponding phonetic character and with the sample word shown below the target syllable. With the appearance of each phrase, a loudspeaker also played a pre-recorded sound file of that phrase spoken by a young adult woman (a native speaker of English and a graduate student in speech-language pathology). After reading the phrase on the monitor and simultaneously hearing the sound file, the subject then produced the same utterance.

A two-dimensional EMA system (Carstens AG200) was used to transduce movements of the tongue and jaw. This recording device limits the placement of all movement sensors (receiver coils) to the midsagittal plane. A cyanoacrylate adhesive (Cyanodent, Ellman International) was used to attach three receiver coils to the tongue: the first one (T1) was positioned ~1 cm from the tip, and the second and third one (T2, T3) were placed ~1.5 and 3 cm more posterior. The same adhesive was also used to attach the jaw sensor (J) to the mandibular gums below the lower central incisors. To allow offline corrections for head movement relative to the articulograph “helmet,” two reference sensors were attached to the bridge of the nose and the maxillary gums above the upper central incisors (with double-sided adhesive tape and Cyanodent, respectively).

At the end of the recording session, subjects kept a bite plate between their upper and lower teeth. The bite plate had two sensors on its top surface, and was inserted such that one sensor was at the facial surface of the tip of the upper incisors and the second sensor was 4 cm into the mouth. Recording these bite plate sensors together with the two reference sensors on the bridge of the nose and maxillary gums allowed us to re-express all data offline from the original helmet-based coordinate system into an anatomically-defined coordinate system in which the x-axis lies in the individual subject's occlusal plane and the origin is at the tip of the upper incisors (Okadome and Honda, 2001; Perkell and Zandipour, 2002; Max et al., 2003).

### Data extraction and analysis

#### Data processing and measurement

Offline data processing was accomplished using custom Matlab routines (The MathWorks, Natick, MA). The kinematic signals were first low-pass filtered with a cut-off frequency of 15 Hz for the sensors on moving articulators and 5 Hz for the stationary reference sensors (Lucero et al., 1997; Green et al., 2000, 2002). Next, the signals from the moving articulators were corrected for head movement (i.e., their coordinates were calculated relative to the stationary reference sensors), and then transformed into the above described anatomically-defined coordinate system. Lastly, all movement paths for a given combination of subject, consonant, and syllable type were shifted such that they had a common starting point with coordinates corresponding to the average *x* and *y* coordinates of all movement starting points across the high, mid, and low vowels produced in that particular condition^1^. For the anterior tongue movements associated with the alveolar consonants /t/ and /s/, data from sensor T1 were used for all measurements; for the more posterior tongue movements associated with the velar consonant /k/, data from sensor T3 were used. Jaw movement data were analyzed for all consonants.

Based on the work by Messier and Kalaska (1999), we measured peak tangential acceleration, peak tangential velocity, movement extent, and the spatial coordinates at peak tangential acceleration, peak tangential velocity, and movement endpoint. Custom Matlab routines first searched for a peak in the tangential velocity profile, and the immediately preceding and following local minima in this tangential velocity signal were automatically considered to define the movement's start and end times, respectively. When there was no clear local minimum in the tangential velocity signal, the acceleration profile was used to determine the start and end times. A tangential acceleration peak in the expected time region indicates that the tangential velocity changed more or less suddenly, and, thus, we interpreted that moment in time as the movement's start or end time.

Peak tangential acceleration was defined as the maximum acceleration value in the time window from the movement's start time to its peak tangential velocity. Movement extent was defined as the straight-line distance from start point to end point (although jaw movements show a relatively straight trajectory, tongue movements are often curved; we therefore also analyzed our data with movement extent measured as the actual distance traveled rather than the straight-line distance, but the two different procedures did not affect the overall outcome of the analyses). The tongue and jaw's spatial coordinates at the time of peak acceleration, peak velocity, and movement endpoint were measured as their (*x, y*) coordinates relative to the anatomically-defined coordinate system.

#### Data analysis

The first step of the data analysis stage involved calculating, for each individual subject and each combination of articulator, syllable type, and consonant, the Pearson correlation coefficients among the three kinematic variables peak acceleration, peak velocity, and movement extent. These correlation coefficients were calculated once across the three vowels paired with a given consonant (thus when movements were specifically planned to achieve three different distances) and, separately, within each individual vowel (thus when all movements were planned to achieve a similar distance).

Second, the articulators' spatial coordinates (*x, y*) were measured at the time of peak tangential acceleration, peak tangential velocity, and movement endpoint to determine the strength of the relationship between early and final positions. The curved trajectories of some articulatory movements, however, made it problematic to calculate correlation coefficients (e.g., between *y* values at the time of peak acceleration and at the time of movement endpoint) directly from the position measures expressed relative to a head-based coordinate system. The tongue tip, for example, may be moving in a different direction at the time of peak acceleration than at the time of peak velocity or at the end of the movement. Thus, instead, we determined—again for each individual subject and for each combination of articulator, syllable type, and consonant—the articulator's spatial coordinates at the three time points of interest relative to the distribution of all equivalent data points across trials; once again including all trials across vowels and then also for each vowel separately.

This analysis was accomplished by first drawing for each cluster of data points (e.g., a given subject's tongue tip positions at peak acceleration for all CV syllables with onset /t/) ellipses that included 95% of the data points and whose major and minor axes were determined by the first two eigenvectors of the covariance matrix of the (*x, y*) coordinates. We then re-expressed all data points relative to the major and minor axes of the corresponding ellipse, and these coordinates were used to calculate the correlation coefficients among position at peak acceleration, position at peak velocity, and position at movement endpoint. The correlation coefficients were computed separately for position relative to the ellipses' major and minor axes as well as for data pooled across vowels of different height and within each category of vowel height.

Visual inspection of the data showed that the major axis of most ellipses was aligned with the movement direction at the corresponding time point. Most important for the present analyses, the major axes of the three ellipses for a given combination of subject, articulator, and consonant (i.e., trials for which the correlations among position at peak acceleration, peak velocity, and endpoint were calculated) were generally aligned. Table 1 lists for how many of the analyzed pairs of ellipses the major axes were aligned to within ±60°. We did not exclude the correlation coefficients calculated from the relatively small number of pairs of ellipses that exceeded this criterion.

**Table 1:** **Ratio of pairs of ellipses with major axes aligned within ±60° to total number of pairs of ellipses**.

	**Jaw**			**Tongue**		
	**A-V**	**V-E**	**A-E**	**A-V**	**V-E**	**A-E**
**ACROSS VOWELS**						
/t/ + V	5/5	5/5	5/5	4/5	5/5	4/5
/s/ + V	5/5	5/5	5/5	5/5	5/5	5/5
V + /t/	5/5	5/5	5/5	5/5	5/5	4/5
V + /s/	5/5	5/5	5/5	5/5	5/5	5/5
**WITHIN EACH VOWEL**						
/t/ + V	14/15	15/15	12/15	12/15	13/15	14/15
/s/ + V	14/15	15/15	14/15	13/14	14/14	12/14
V + /t/	14/15	14/15	14/15	12/14	12/14	9/14
V + /s/	14/15	15/15	13/15	12/14	13/14	12/14

The total number of pairs of ellipses in each cell is 1 per subject in the “across vowels” case and 3 per subject in the “within each vowel” case (high, mid, low vowels separately) minus combinations with insufficient data (see text). A, ellipse with acceleration coordinates; V, ellipse with velocity coordinates; E, ellipse with endpoint coordinates.

There were also some cases in which the cluster of (*x, y*) data points (mostly at the time of peak acceleration) did not have an elliptical shape. If the ratio of the length of the minor axis to the length of the major axis was greater than 0.65 (i.e., if the shape was circular or approximately circular), we first empirically determined the best possible alignment of such a data cluster with the elliptical ones at the remaining time points (mostly at the time of peak velocity and movement endpoint). In these exceptional cases where the “true” orientation of major and minor axis could not be determined, we repeatedly rotated the eigenvalues-based coordinate system in steps of 1/6th of a degree to find, for the major axis projections, the maximum correlation with the data clusters at the remaining time points. All Pearson correlation coefficients were then calculated based on the rotated coordinate system.

As a third analysis method, a statistical model was used to explore the possibility of estimating the strength of specific feedback contributions. The basic idea is that such contributions can be inferred when the following conditions are met: (a) peak acceleration and peak velocity scale with movement extent for movements across as well as within different targets, (b) for movements toward the same target, another dependent variable correlates negatively with peak acceleration and/or peak velocity, and (c) the addition of this other variable into a multiple correlation model causes a statistically significant increase in the proportion of explained variance for movement extent as compared with that explained by the bivariate correlation between peak acceleration or velocity and movement extent alone (Gordon and Ghez, 1987). For example, online adjustments in movement duration^2^ may be used to compensate for variability in the achieved peak acceleration or velocity (Ostry and Munhall, 1985; Messier and Kalaska, 1999). In this case, the coefficient of determination for the bivariate relationship between either peak acceleration or peak velocity and movement extent can be considered to estimate the strength of feedforward contributions, whereas the coefficient of multiple determination that is obtained after adding movement duration as an additional predictor variable quantifies the proportion of variance in movement extent that is accounted for by the *combination* of feedforward contributions and feedback contributions that involved adjustments in movement duration.

Hereafter, we refer to the bivariate coefficient of determination as *r*^2^_*Y*|*X1*_, where *r*^2^ is the squared correlation coefficient for the variables *Y* and *X1*; *Y* is movement extent and *X1* is peak acceleration or peak velocity. We refer to the coefficient of multiple determination as *R*^2^_*Y*| *X*1,*X*2_, where Y, X1, and X2 are the variables movement extent, peak acceleration or peak velocity, and movement duration, respectively. This coefficient of multiple determination was calculated for each individual subject and each combination of articulator, syllable type, and consonant as follows (Kleinbaum and Kleinbaum, 1998):

$$R_{Y|X1,X2}^{2}=1-\frac{\sum_{i\;=\;1}^{n}{\left(Y_{i}-{\hat{Y}}_{i}\right)}^{2}}{\sum_{i\;=\;1}^{n}{\left(Y_{i}-\bar{Y}\right)}^{2}}$$

where *Y*_*i*_ is movement extent for trial *i* and $\hat{Y}$_*i*_ is the value of *Y*_*i*_ predicted by the combination of *X1*_*i*_ and *X2*_*i*_. $\hat{Y}$_*i*_ is obtained from the following least-squares solution:

$${\hat{Y}}_{i}={\hat{\beta }}_{0}+{\hat{\beta }}_{1}X1_{i}+{\hat{\beta }}_{2}X2_{i}$$

To limit the number of additional tests, these coefficients were calculated only across the different vowels and not within each vowel separately.

The differences *R*^2^_*Y*|*X*1,*X*2_ − *r*^2^_*Y*|*X*1_ were then calculated as estimates of the proportion of variance in movement extent that could be attributed to feedback-driven adjustments in movement duration. In addition to deriving those descriptive estimates, an *F* statistic was calculated to assess whether the variance explained by *R*^2^_*Y*|*X*1,*X*2_ was statistically significantly greater than that explained by *r*^2^_*Y*|*X*1_ (Sokal and Rohlf, 1981):

$$F=\frac{\left(R_{Y|X1,X2}^{2}-r_{Y|X1}^{2})/(k_{R^{2}}-k_{r^{2}}\right)}{\left(1-R_{Y|X1,X2}^{2})/(n-k_{R^{2}}-1\right)}$$

where *Y*, *X1*, and *X2* are as described above, *k* is the number of variables included to predict *Y* (i.e., 2 for *k*_*R*^2^_, 1 for *k*_*r*^2^_), and *n* is the number of trials used in the calculation of the coefficients of determination. This *F* statistic was tested at α = 0.05 with (*k*_*R*^2^_ − *k*_*r*^2^_, *n* − *k*_*R*^2^_ − 1) degrees of freedom (Sokal and Rohlf, 1981).

### Data selection

Similar to the reaching movement paradigm of Messier and Kalaska (1999), we aimed to elicit movements with different target distances. For this purpose, we included high, mid, and low vowels in the target syllables. Preliminary analyses showed that this attempt to elicit movements of different amplitude was generally successful, but not in all cases. In particular, even with uncorrected α levels, trials with consonant /k/ (articulated by bringing the tongue dorsum in contact with the velum) failed to result in significantly different T3 amplitudes across vowels for several combinations of articulator and syllable type [jaw for mid vs. low back V in VC, *t*_(4)_ = −0.594, *p* = 0.585; tongue for mid vs. low back V in CV, *t*_(4)_ = 2.205, *p* = 0.092; tongue for mid vs. low back V in VC, *t*_(4)_ = 1.267, *p* = 0.274; jaw for mid vs. low front V in VC, *t*_(4)_ = −2.757, *p* = 0.051; tongue for mid vs. low front V in VC, *t*_(4)_ = −2.572, *p* = 0.062]. This was much less often the case for consonant /s/ [tongue for mid vs. low back V in CV, *t*_(4)_ = 0.292, *p* = 0.785] or consonant /t/ [jaw for mid vs. low back V in CV, *t*_(4)_ = −2.248, *p* = 0.088; jaw for mid vs. low back V in VC, *t*_(4)_ = −0.551, *p* = 0.611], and in those cases only trials with back vowels were affected. Thus, given that the underlying rationale assumed the inclusion of movements with different target distances, the data set selected for statistical analysis included all productions with the consonants /t/ or /s/ and the front vowels /i, ε, æ/. This data set included both tongue and jaw movement data for a total of 804 utterances across the five participants.

As expected, some movements showed multiple peaks (rather than a single peak) in the tangential velocity profile. Double peaks in the tangential velocity profile are particularly common in movements with curvilinear trajectories (Abend et al., 1982; Quinn et al., 1997). For the present purposes, such trials with multiple peaks in the velocity signal had to be discarded because the dependent variables of interest could not be unambiguously extracted when there were multiple velocity peaks. In the present data set, 7.59% of all jaw movements and 11.44% of all tongue movements were problematic in this regard. Combined with other reasons for data exclusion (e.g., production errors not noticed during the recording session), this led to three cases in which a given subject contributed fewer than 4 usable trials for a given combination of articulator, syllable type, consonant, and vowel (e.g., subject S3's tongue data for CV syllables in which the consonant /s/ was combined with the high vowel /i/). In those cases, the subject was not included in the analyses for that specific combination of conditions. The number of analyzed vs. excluded trials for each subject and each condition is listed in Table 2.

**Table 2:** **Ratio of number of available trials to number of recorded trials**.

	**/ti/**		**/tε/**		**/tæ/**		**/si/**		**/sε/**		**/sæ/**	
	**J**	**T**	**J**	**T**	**J**	**T**	**J**	**T**	**J**	**T**	**J**	**T**
S1	10/12	9/12	12/12	11/12	8/12	7/12	10/12	9/12	12/12	12/12	12/12	12/12
S2	13/15	6/15	13/15	9/15	13/15	11/15	15/15	13/15	15/15	14/15	15/15	15/15
S3	13/15	6/15	15/15	14/15	14/15	12/15	8/15	*3/15*	15/15	15/15	14/15	14/15
S4	4/15	5/15	15/15	15/15	15/15	10/15	9/15	8/15	15/15	14/15	15/15	15/15
S5	10/10	8/10	9/10	9/10	10/10	9/10	5/10	7/10	9/10	9/10	10/10	9/10
	**/it/**		**/εt/**		**/æt/**		**/is/**		**/εs/**		**/æs/**	
	**J**	**T**	**J**	**T**	**J**	**T**	**J**	**T**	**J**	**T**	**J**	**T**
S1	10/12	12/12	12/12	12/12	12/12	12/12	11/12	12/12	12/12	12/12	12/12	12/12
S2	15/15	15/15	15/15	15/15	14/15	14/15	15/15	15/15	15/15	15/15	15/15	15/15
S3	12/15	*2/15*	15/15	15/15	15/15	15/15	6/15	*2/15*	15/15	15/15	15/15	15/15
S4	6/15	12/15	15/15	15/15	15/15	15/15	11/15	12/15	15/15	15/15	15/15	15/15
S5	8/10	8/10	10/10	10/10	9/10	7/10	8/10	7/10	9/10	9/10	10/10	9/10

Italicized data indicate combinations of subject and condition that were excluded from analysis because less than 4 trials were available. S1 … S5 denote the individual subjects. J, jaw; T, tongue.

## Results

### General observations

**Figure 1:**
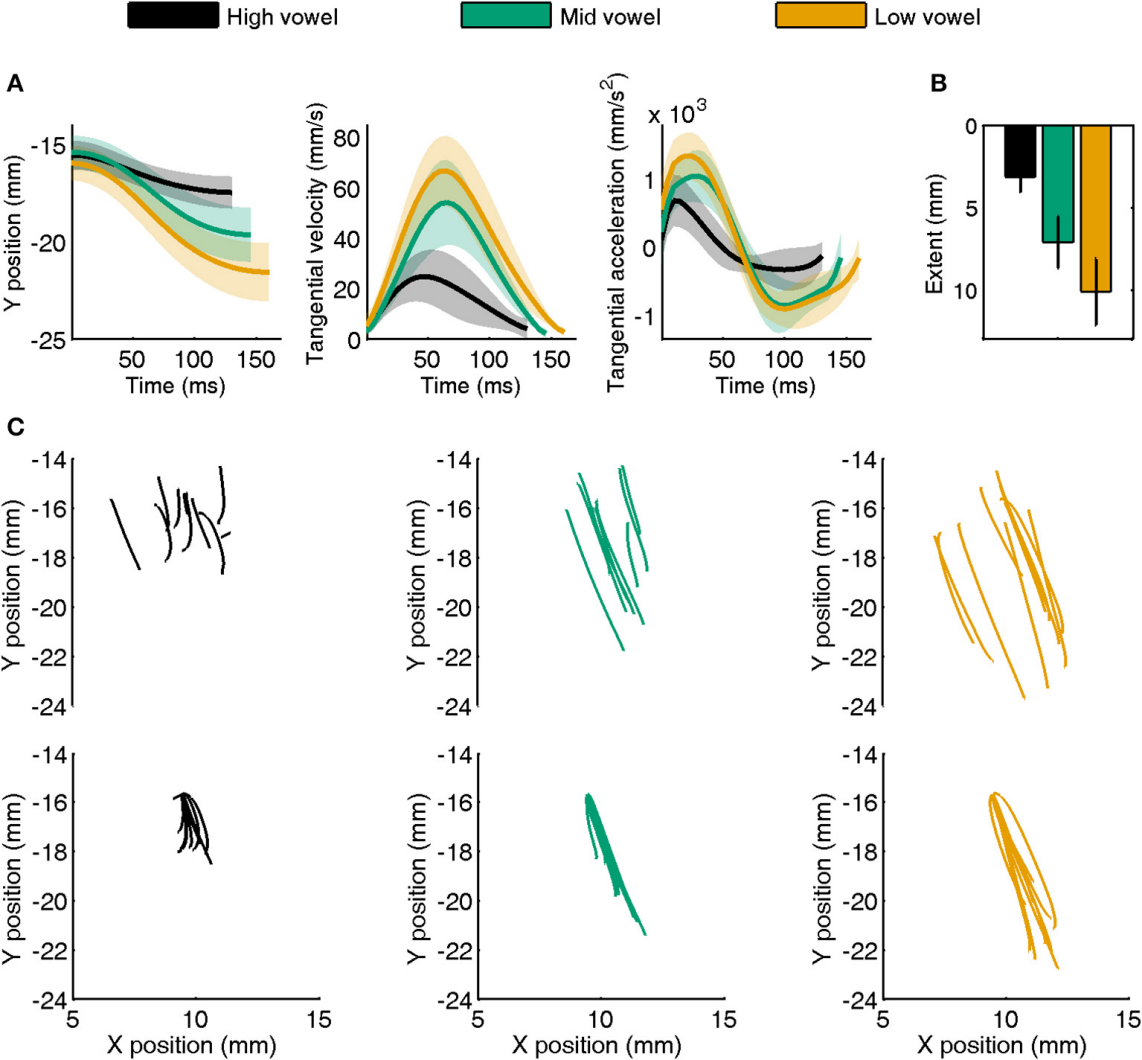
**(A)** Example of a single subject's jaw movement data for CV syllables with consonant /t/. Separate panels show the time series of *y*-axis position, tangential velocity, and tangential acceleration averaged for high, mid, and low vowels (shaded areas indicate *SE*). Average data were obtained after normalizing movement duration within vowel category. **(B)** Group mean (with *SE*) movement extent for high, mid, and low vowels in the same target syllables. **(C)** Individual trial movement paths for the same data as shown in **(A)**. The top half of each panel shows raw data whereas the lower panels show all paths aligned to the same starting point.

Representative jaw movements from a single subject's CV trials with consonant /t/ are illustrated in Figure 1A. The tangential velocity profiles were generally bell-shaped, and they scaled with movement extent. Similarly, the tangential acceleration profiles showed typical acceleration and deceleration phases, and acceleration also scaled with movement extent. Both the individual subject data in Figure 1A and the group mean data for jaw extent in Figure 1B confirm that, for these front vowels, the speech task achieved its goal of eliciting movements that differed in extent across the three categories of vowel height. Although not included in Figure 1, tongue movements generally followed the same pattern, with the exception that more tongue movement paths were curved rather than straight (but the degree of curvature was not quantified for the purpose of the present study).

Figure 1C is based on the same single subject /t/ + V jaw data shown in Figure 1A. The three upper panels illustrate that the starting points of individual trial motion paths were highly variable even within each vowel category (identical CV syllables). The three bottom panels show the effect of our post-processing procedure that aligned all paths to the same starting point (corresponding to the average (*x*, *y*) coordinates across the subject's trials for this combination of articulator, syllable type, and consonant) to eliminate the confounding influence of varying starting points on our correlational analyses with the spatial coordinates of early vs. late kinematics (see Footnote 1).

### Correlation between peak acceleration or peak velocity and movement extent

**Figure 2:**
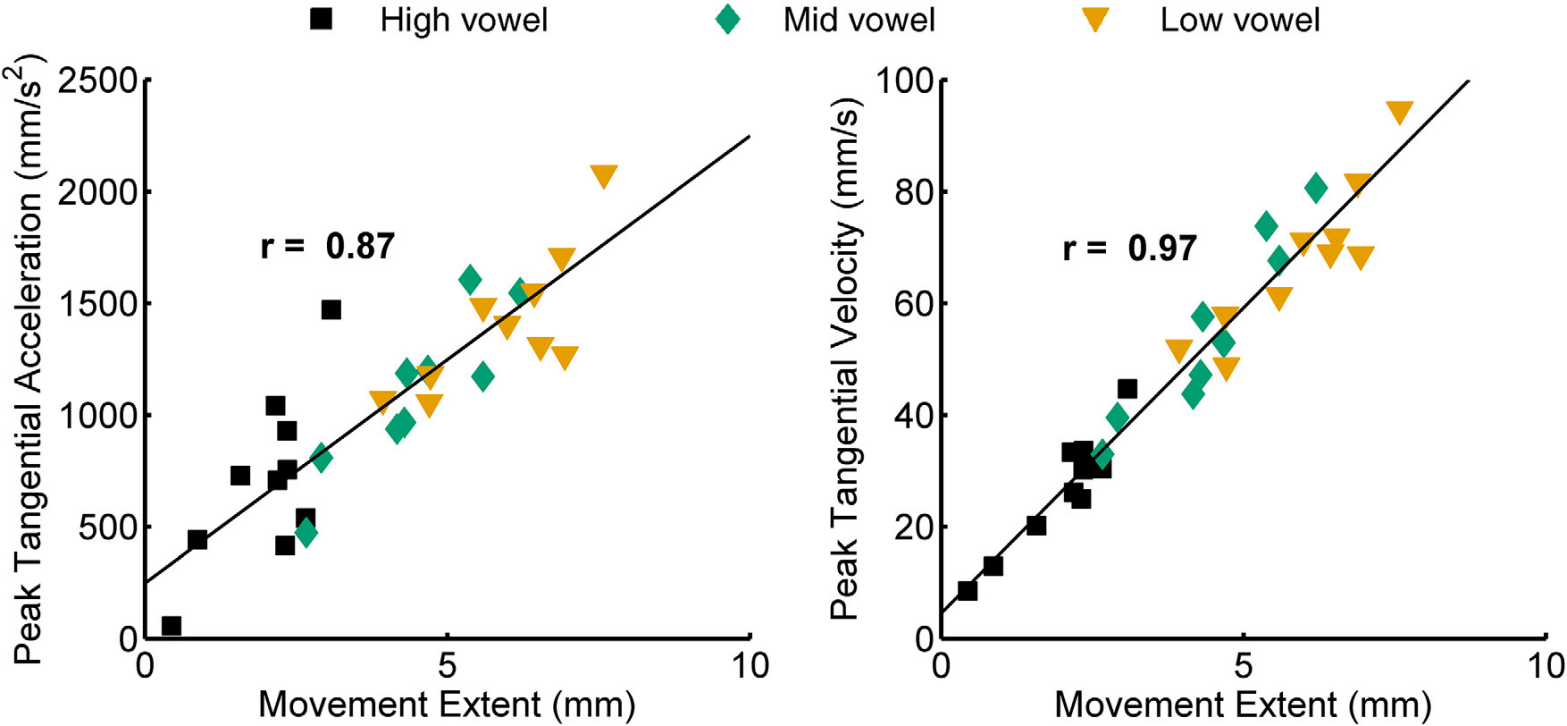
**Example of a single subject's data for the correlation between movement extent and peak acceleration (left) and movement extent and peak velocity (right)**. Data shown are for jaw movements in CV syllables with consonant /t/.

**Figure 3:**
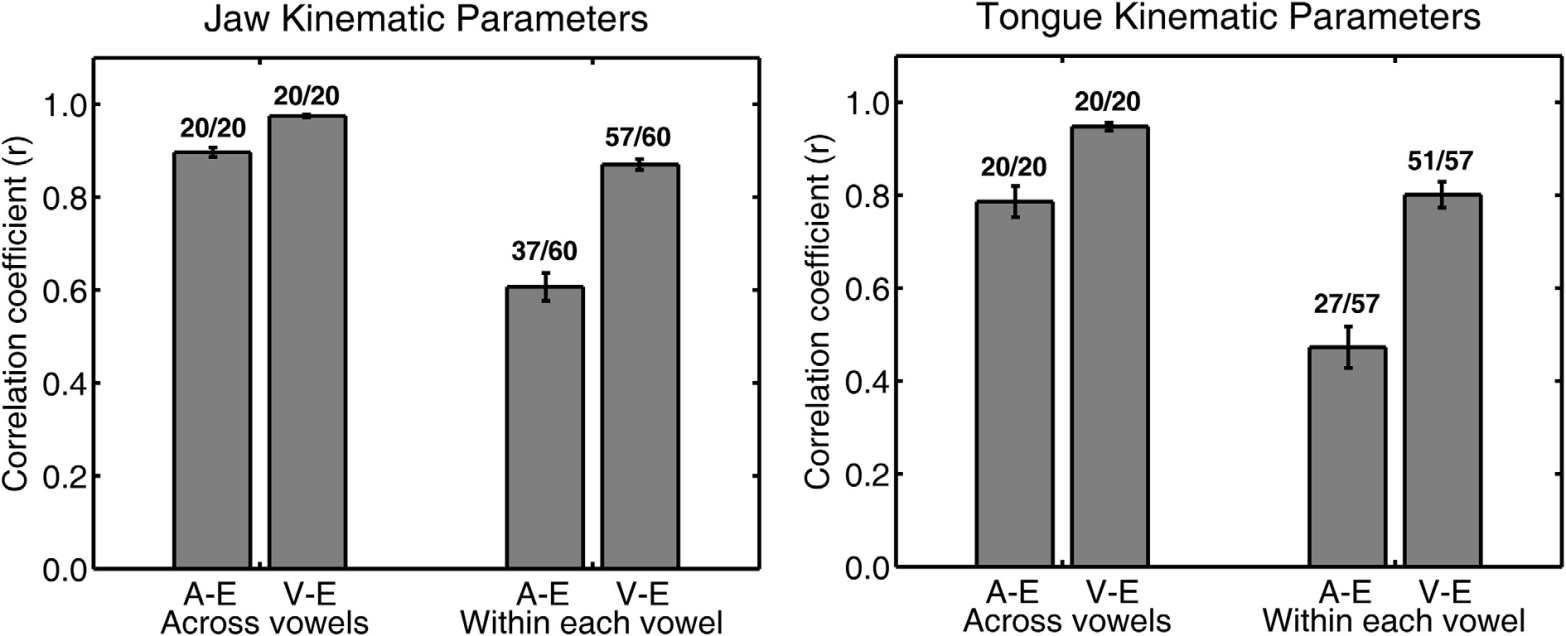
**Group mean (with *SE* error bars) Pearson correlation coefficients for jaw (left panel) and tongue (right panel) peak acceleration (A) or peak velocity (V) and movement extent (E) across all four syllable combinations**. Numbers above each bar indicate the ratio of statistically significant correlation coefficients (*p* < 0.05) to the total number of correlation coefficients. The total number of correlation coefficients is 5 subjects × 4 syllables for “across vowels” analyses and 5 subjects × 4 syllables × 3 vowels for “within each vowel” analyses.

Figure 2 shows an example of single subject jaw data (same subject as in Figure 1) for the correlation between peak acceleration or peak velocity and movement extent. The group mean correlation coefficients for all combinations of syllable type and consonant are listed in the upper part of Table 3 for jaw data and in the upper part of Table 4 for tongue data. Figure 3 graphically illustrates the overall mean correlation data across those conditions as well as the proportion of individual subject correlation coefficients that were statistically significant (across vowels: 5 subjects × 2 syllable types × 2 consonants; within each vowel: 5 subjects × 2 syllable types × 2 consonants × 3 vowels).

**Table 3:** **Pearson correlation coefficients (*M*, group mean; *SE*, standard error of the mean) for jaw movement early and late kinematics**.

		**Jaw**							
		**Across vowels**				**Within each vowel**			
		**A-E**		**V-E**		**A-E**		**V-E**	
		***M***	***SE***	***M***	***SE***	***M***	***SE***	***M***	***SE***
**KINEMATIC VALUES**									
	/t/ + V	0.8799	0.0222	0.9636	0.0079	0.6323	0.0417	0.8197	0.0279
	/s/ + V	0.8979	0.0179	0.9671	0.0038	0.6495	0.0404	0.8774	0.0154
	V + /t/	0.9089	0.0184	0.9834	0.0041	0.6244	0.0653	0.9167	0.0136
	V + /s/	0.9002	0.0255	0.9849	0.0028	0.5213	0.0835	0.8665	0.0260
**SPATIAL COORDINATES**									
Major axis	/t/ + V	0.7400	0.0616	0.9653	0.0057	0.4762	0.0587	0.8404	0.0366
Major axis	/s/ + V	0.7672	0.0671	0.9755	0.0080	0.4585	0.0882	0.8946	0.0227
Major axis	V + /t/	0.7603	0.0487	0.9786	0.0047	0.5689	0.0537	0.9198	0.0130
Major axis	V + /s/	0.7791	0.0511	0.9702	0.0159	0.5824	0.0667	0.8916	0.0192
Minor axis	/t/ + V	0.3240	0.0785	0.7179	0.0350	0.3559	0.0988	0.6316	0.0506
Minor axis	/s/ + V	0.5384	0.0532	0.8063	0.0503	0.3278	0.0824	0.6784	0.0758
Minor axis	V + /t/	0.4022	0.0690	0.7916	0.0433	0.4138	0.0696	0.7586	0.0514
Minor axis	V + /s/	0.2880	0.1862	0.7379	0.0806	0.2187	0.0893	0.6865	0.0450

In the upper 4 rows, A-E refers to the correlation between peak acceleration and extent, V-E refers to the correlation between peak velocity and extent. In the lower 8 rows, A-E refers to the correlation between spatial coordinates at peak acceleration and endpoint, V-E refers to the correlation between spatial coordinates at peak velocity and endpoint. Correlations for each subject were calculated based on all trials across three categories of vowel height or as the average of the correlation coefficients based on trials within each vowel category separately.

**Table 4:** **Pearson correlation coefficients (*M*, group mean; *SE*, standard error of the mean) for tongue movement early and late kinematics**.

		**Tongue**							
		**Across vowels**				**Within each vowel**			
		**A-E**		**V-E**		**A-E**		**V-E**	
		***M***	***SE***	***M***	***SE***	***M***	***SE***	***M***	***SE***
**KINEMATIC VALUES**									
	/t/ + V	0.7284	0.0782	0.9272	0.0254	0.2376	0.1202	0.6721	0.0912
	/s/ + V	0.8323	0.0393	0.9477	0.0106	0.6109	0.0439	0.8482	0.0215
	V + /t/	0.7522	0.0972	0.9452	0.0201	0.4685	0.0779	0.8479	0.0306
	V + /s/	0.8313	0.0452	0.9703	0.0077	0.5899	0.0581	0.8452	0.0322
**SPATIAL COORDINATES**									
Major axis	/t/ + V	0.5191	0.0955	0.8880	0.0374	0.1898	0.1476	0.7093	0.1098
Major axis	/s/ + V	0.7664	0.0581	0.9628	0.0100	0.6201	0.0496	0.9130	0.0178
Major axis	V + /t/	0.8659	0.0204	0.9835	0.0024	0.7097	0.0482	0.9328	0.0161
Major axis	V + /s/	0.7762	0.0910	0.9780	0.0046	0.5811	0.0921	0.9273	0.0119
Minor axis	/t/ + V	0.4545	0.1081	0.7563	0.0788	0.2016	0.1049	0.6259	0.0851
Minor axis	/s/ + V	0.5312	0.0888	0.8384	0.0477	0.2320	0.1334	0.8064	0.0260
Minor axis	V + /t/	0.4923	0.1171	0.7275	0.0337	0.5269	0.1033	0.7947	0.0325
Minor axis	V + /s/	0.4966	0.0813	0.8057	0.0348	0.4066	0.0642	0.7793	0.0430

In the upper 4 rows, A-E refers to the correlation between peak acceleration and extent, V-E refers to the correlation between peak velocity and extent. In the lower 8 rows, A-E refers to the correlation between spatial coordinates at peak acceleration and endpoint, V-E refers to the correlation between spatial coordinates at peak velocity and endpoint. Correlations for each subject were calculated based on all trials across three categories of vowel height or as the average of the correlation coefficients based on trials within each vowel category separately.

For both jaw and tongue movements, the correlations between peak acceleration or peak velocity and movement extent were positive and generally high. Correlations between peak acceleration and extent were descriptively lower than those between peak velocity and extent, but this was fully expected as peak acceleration occurs very early in the movement whereas peak velocity occurs approximately in the middle of the movement. In fact, given how early in the movement peak acceleration is reached (Figure 1A), the strong relationship between peak acceleration and movement extent is noteworthy. For example, when calculated based on jaw movements to different vowel targets in otherwise identical syllables (“across vowels”), not only peak velocity but also peak acceleration was statistically significantly correlated with movement extent in each of the 20 cases (Figure 3, left panel). For the jaw, the group average correlations between peak acceleration and extent were in the range 0.88–0.91 for the four different syllable combinations (/t/ + V, /s/ + V, V + /t/, V + /s/) whereas those between peak velocity and extent were in the range 0.96–0.98. Similarly, for tongue movements to different vowel targets in otherwise identical syllables, the correlation coefficients for both peak velocity vs. movement extent and for peak acceleration vs. movement extent were also statistically significant in all 20 cases (Figure 3, right panel). For the tongue, the group average correlations between peak acceleration and extent were in the range 0.73–0.83 for the different syllable combinations whereas those for peak velocity and extent were in the range 0.93–0.97. Overall, these correlations between peak acceleration or peak velocity and movement extent for jaw and tongue movements to different targets are in the same range as those reported for arm movements when reaching to visual targets at different distances (Messier and Kalaska, 1999).

When calculated based on jaw movements from trials that all included the same target syllable with the same target vowel (“within each vowel”), the group mean correlations with movement extent were reduced more substantially for peak acceleration than for peak velocity. Nevertheless, the within-vowel correlations with extent were still in the range 0.52–0.65 for peak acceleration and 0.82–0.92 for peak velocity. Accordingly, for these within-vowel analyses, the proportion of correlation coefficients that was statistically significant dropped to 61.67% for peak acceleration but only to 95.00% for peak velocity (Figure 3, left panel). Such a reduction in the strength of the observed relationships was also noticeable in the tongue data where the proportion of statistically significant correlation coefficients in the within-vowel analyses dropped to 47.37% for peak acceleration and 89.47% for peak velocity (Figure 3, right panel). Consequently, the tongue group mean correlation coefficients were also substantially lower here than for the across-vowels analyses: 0.24–0.61 for peak acceleration vs. extent and 0.67–0.85 for peak velocity vs. extent (Table 4). Interestingly, for both the jaw and the tongue, these correlation coefficients based on movements aiming for the same target vowel are descriptively higher than those available for limb reaching movements aiming for a single target distance (Messier and Kalaska, 1999).

### Correlation between spatial coordinates at peak acceleration or peak velocity and at movement endpoint

**Figure 4:**
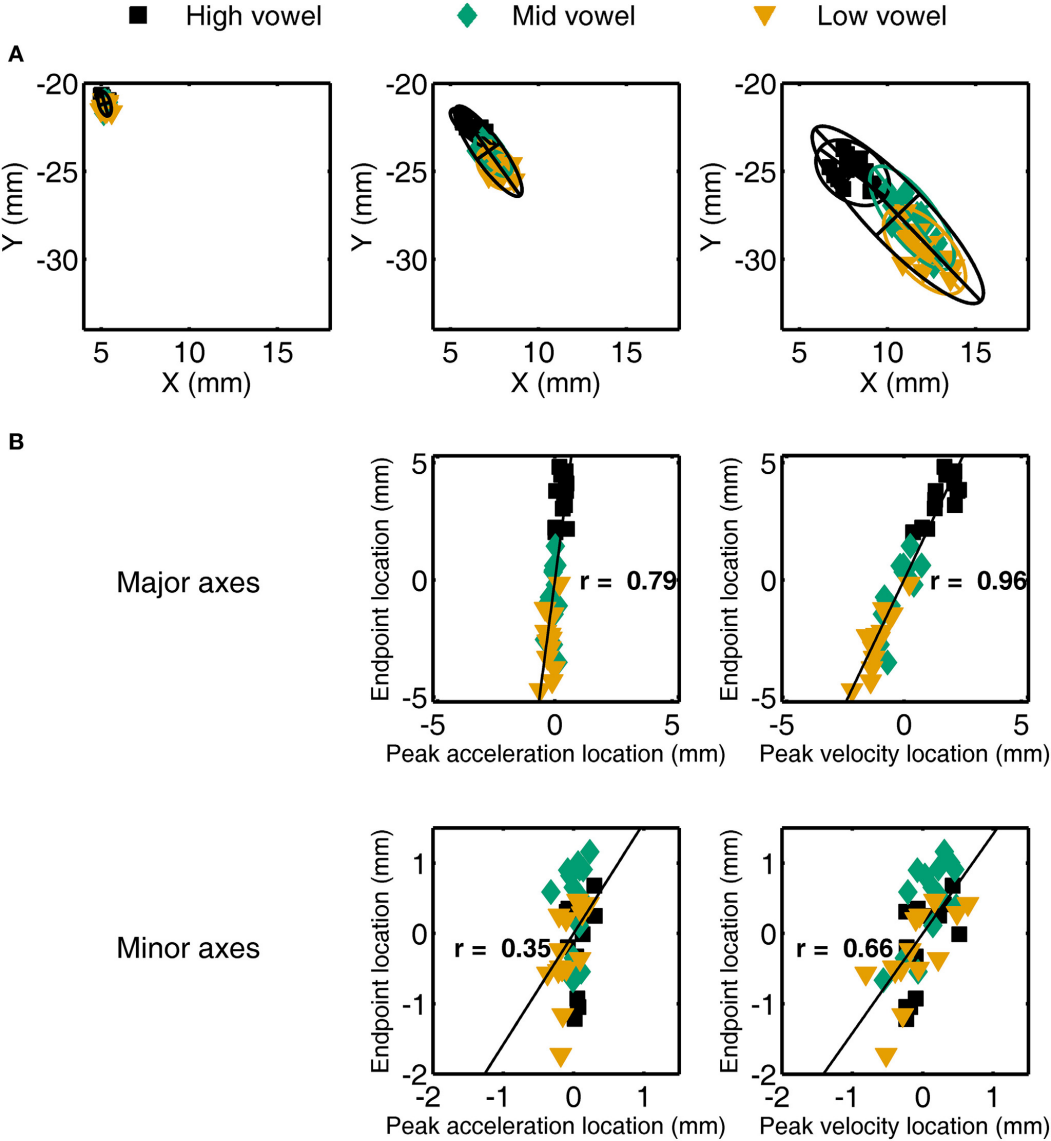
**(A)** Illustration of jaw spatial coordinates at peak acceleration (left), peak velocity (middle), and movement endpoint (right) for a single subject's /t/ + V syllables. Overlaid ellipses include 95% of the data points with the major and minor axes determined by the first two eigenvectors of the covariance matrix of the (*x, y*) coordinates. **(B)** For the same individual subject data, scatter plots and correlation coefficients for the relationship between spatial coordinates at peak acceleration (left) or peak velocity (right) and at movement endpoint after re-expressing those locations relative to the major and minor axes of the ellipses shown in **(A)**. Data expressed relative to the ellipse's major axis are in the upper row, data expressed to the minor axis are in the bottom row.

**Figure 5:**
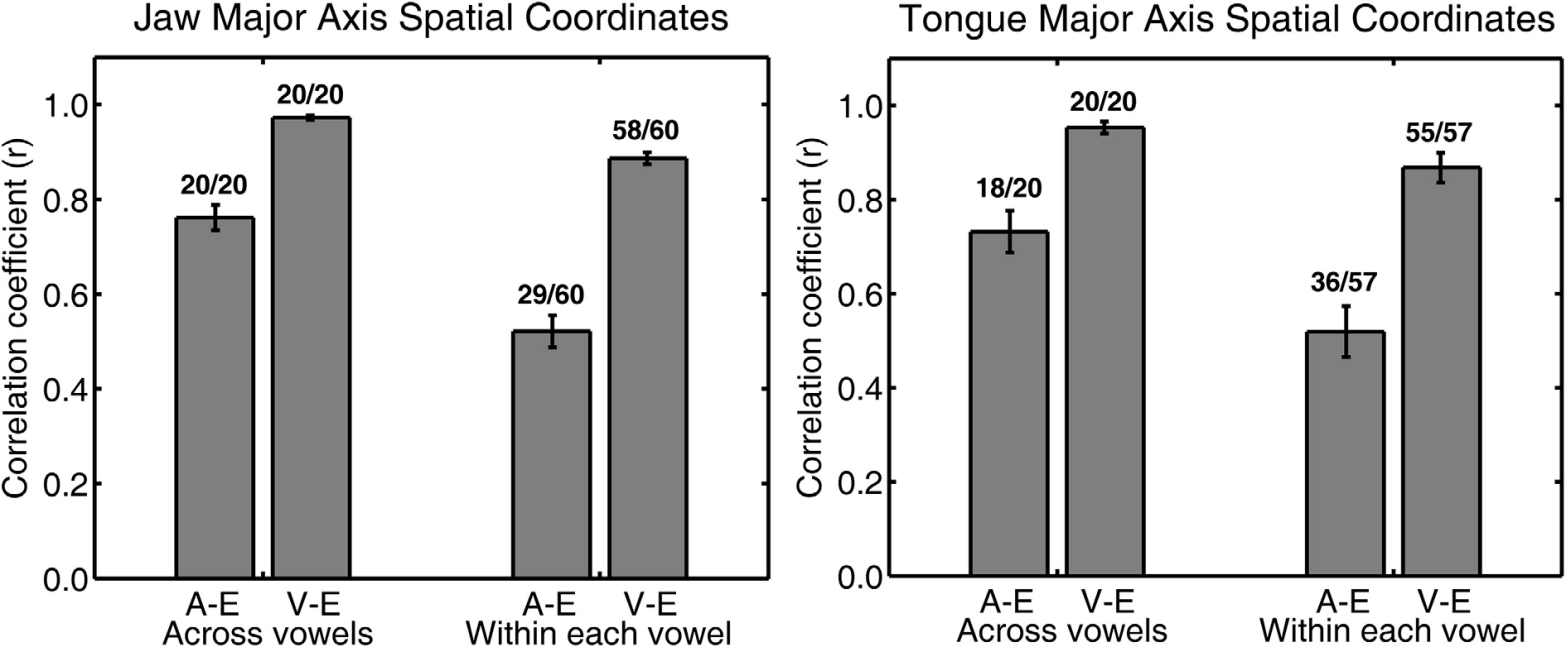
**Group mean (with *SE* error bars) Pearson correlation coefficients for jaw (left panel) and tongue (right panel) spatial coordinates at peak acceleration (A) or at peak velocity (V) and at movement endpoint (E) across all four syllable combinations**. Numbers above each bar indicate the ratio of statistically significant correlation coefficients (*p* < 0.05) to the total number of correlation coefficients. The total number of correlation coefficients is 5 subjects × 4 syllables for “across vowels” analyses and 5 subjects × 4 syllables × 3 vowels for “within each vowel” analyses.

For a single subject's jaw movements in /t/ + V syllables, the spatial coordinates at peak acceleration, peak velocity, and movement endpoint are illustrated in Figure 4A. The corresponding across-vowels correlation coefficients for the coordinates at either peak acceleration or peak velocity and at movement endpoint—with all coordinates expressed to the major and minor axes of their respective 95% ellipses—are shown in Figure 4B. Group data for the across-vowels and within-vowels correlation coefficients are listed in the bottom portion of Table 3 for the jaw and the bottom portion of Table 4 for the tongue. To provide detailed information from the individual subject level, Figure 5 graphically illustrates the proportion of individual subject correlation coefficients that were statistically significant (across vowels: 5 subjects × 2 syllable types × 2 consonants; within each vowel: 5 subjects × 2 syllable types × 2 consonants × 3 vowels) together with the overall mean correlation across syllable combinations. We focus here on the correlations based on major axis coordinates given that those data relate to the intended target distance whereas the minor axis data generally relate to deviations orthogonal to the movement direction.

For jaw movements, the across-vowels correlation coefficients between spatial coordinates at peak velocity and at movement endpoint were again statistically significant in all 20 cases (Figure 5, left panel), and the group means were equally high (0.96–0.98 for the 4 different syllables) as those reported above based on the kinematic parameters peak velocity and movement extent. Group mean correlation coefficients based on the major axis spatial coordinates at peak acceleration and movement endpoint (0.74–0.78) were not as high as those based on the kinematic parameters peak acceleration and movement extent, but all 20 cases were still statistically significant. Tongue movements showed the same pattern: the group mean across-vowels correlation coefficients for spatial coordinates at peak velocity vs. movement endpoint (group means 0.89–0.98 for the different syllables, statistically significant in all 20 cases, Figure 5, right panel) were as high as those for the kinematic parameters peak velocity vs. movement extent, but the group mean across-vowels correlations between spatial coordinates at peak acceleration and movement endpoint (group means 0.52–0.87, statistically significant in 18 of 20 cases) were slightly lower than those for the kinematic parameters peak acceleration and movement extent.

Based on the spatial coordinates data, correlation coefficients for trials that included only the same target syllable with the same target vowel (“within each vowel”) were, as expected, again lower than those calculated across all trials (“across vowels”). For the jaw, the group means for this set of correlation coefficients were 0.84–0.92 for the coordinates at peak velocity vs. movement endpoint and 0.46–0.58 for the coordinates at peak acceleration vs. movement endpoint. Of all the individual correlations with the spatial coordinates at movement endpoint, the proportion that was statistically significant was reduced to 96.67% for the spatial coordinates at peak velocity and 48.33% for the spatial coordinates at peak acceleration (Figure 5, left panel). For tongue movements, the group mean within-each-vowel correlation coefficients based on the spatial data were still high (0.71–0.93) for peak velocity, but the set of four coefficients for peak acceleration contained one very low outlier (0.19 for /t/ + V syllables, 0.62–0.71 for the remaining three syllable combinations). Despite the fact that these analyses were performed with the spatial coordinates data and were based only on movements toward identical vowels, the proportion of individual correlation coefficients that was statistically significant remained as high as 96.49% for peak velocity and 63.16% for peak acceleration (Figure 5, right panel). In fact, these mean correlation coefficients for within-target analyses based on the spatial coordinates data are substantially higher (on the order of 0.40 correlation points) than those reported for arm reaching movements with a single target distance (Messier and Kalaska, 1999).

### Quantification of feedback contributions

**Figure 6:**
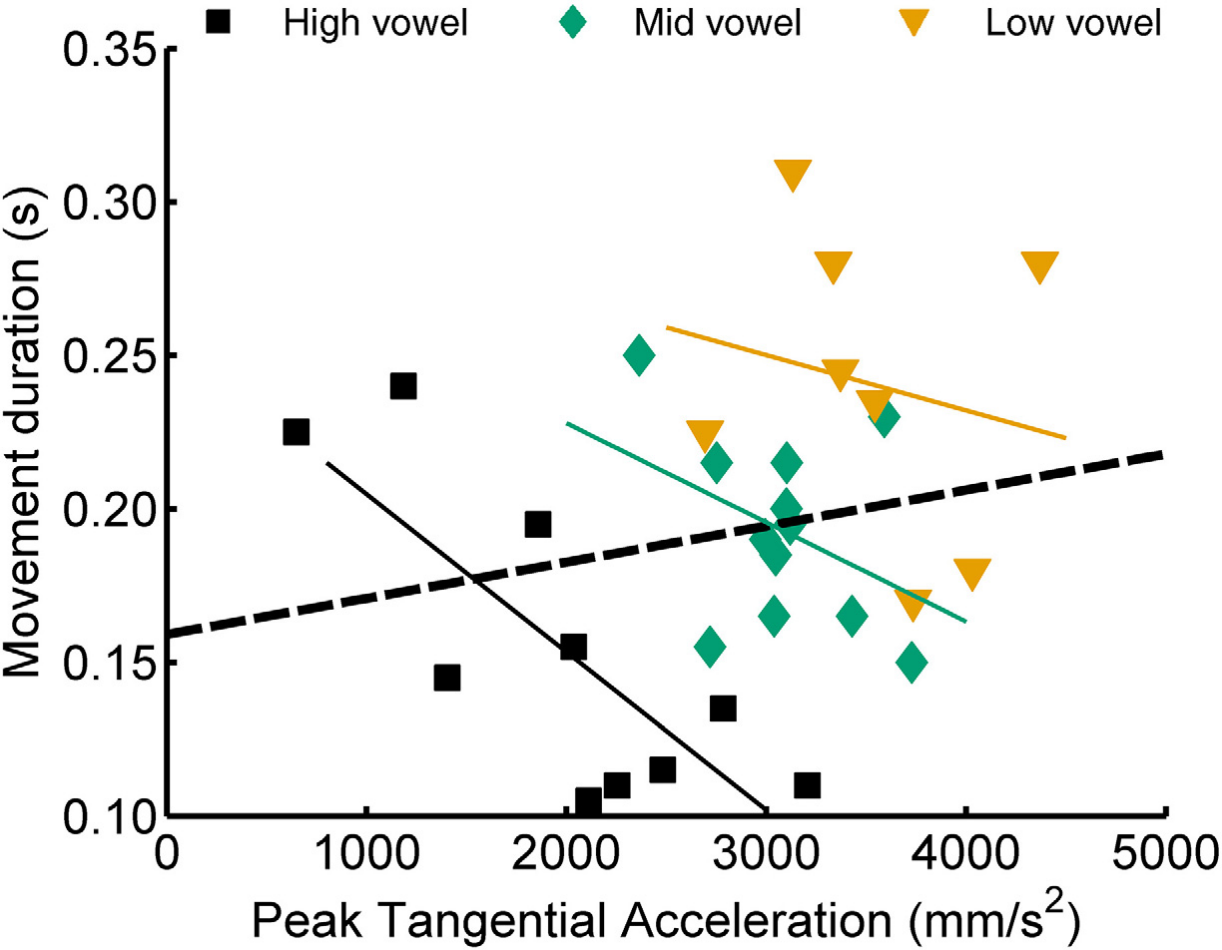
**Example single subject data illustrating a case (jaw movements, CV syllables, consonant /t/) for which the correlation between peak acceleration and movement duration was positive when calculated across the three vowel categories but negative for each of the three within-vowel analyses**.

In their study of reaching movements, Messier and Kalaska (1999) found that movement duration correlated positively with peak acceleration or velocity when analyzing movements across different target distances but that this correlation tended to be negative when analyzing only movements toward the same target. Consequently, those authors concluded that movement duration might be one variable that is adjusted online to compensate for variability in the achieved values of peak acceleration and peak velocity. Interestingly, for the speech data studied here, we obtained a similar result for the correlation between movement duration and peak acceleration but not for movement duration and the later occurring peak velocity. Considering jaw movements, the correlation between duration and peak acceleration was positive in 17/20 cases across vowel height targets (5 subjects × 2 syllable types × 2 consonants) but negative in 41/60 cases within vowel height targets (5 subjects × 2 syllable types × 2 consonants × 3 vowels). Figure 6 shows a single subject's jaw data for one particular case (CV syllables with consonant /t/) where the correlation between peak acceleration and movement duration was positive when calculated across the three vowel categories but negative within each of the three vowel categories separately. For tongue movements, these correlations between duration and peak acceleration were positive in 18/20 cases across vowels but negative in 42/57 cases within vowels (note that the latter number of cases is lower because, for tongue movements, some subjects did not have a sufficient number of usable trials, see Table 2). In fact, of the correlations between duration and peak acceleration that reached statistical significance (uncorrected *p*-values), 95% were positive correlation coefficients for the across vowels analyses but 100% were negative correlation coefficients for the within-vowel analyses. The relationship between movement duration and peak velocity, on the other hand, did not show a clear effect: for the within-vowel analyses, the number of positive and negative correlations was similar, and of the correlations that reached statistical significance, only 54% were negative correlation coefficients.

Given the apparent compensatory adjustments in movement duration based on variation in peak acceleration, we estimated the combination of feedforward contributions plus duration-based feedback contributions by means of the coefficient of multiple determination for peak acceleration, movement extent, and movement duration (or the corresponding spatial coordinates together with movement duration). We estimated the strength of duration-based feedback contributions alone as the difference between this coefficient of multiple determination and the bivariate coefficient of determination for peak acceleration and movement extent (or the corresponding spatial coordinates).

**Figure 7:**
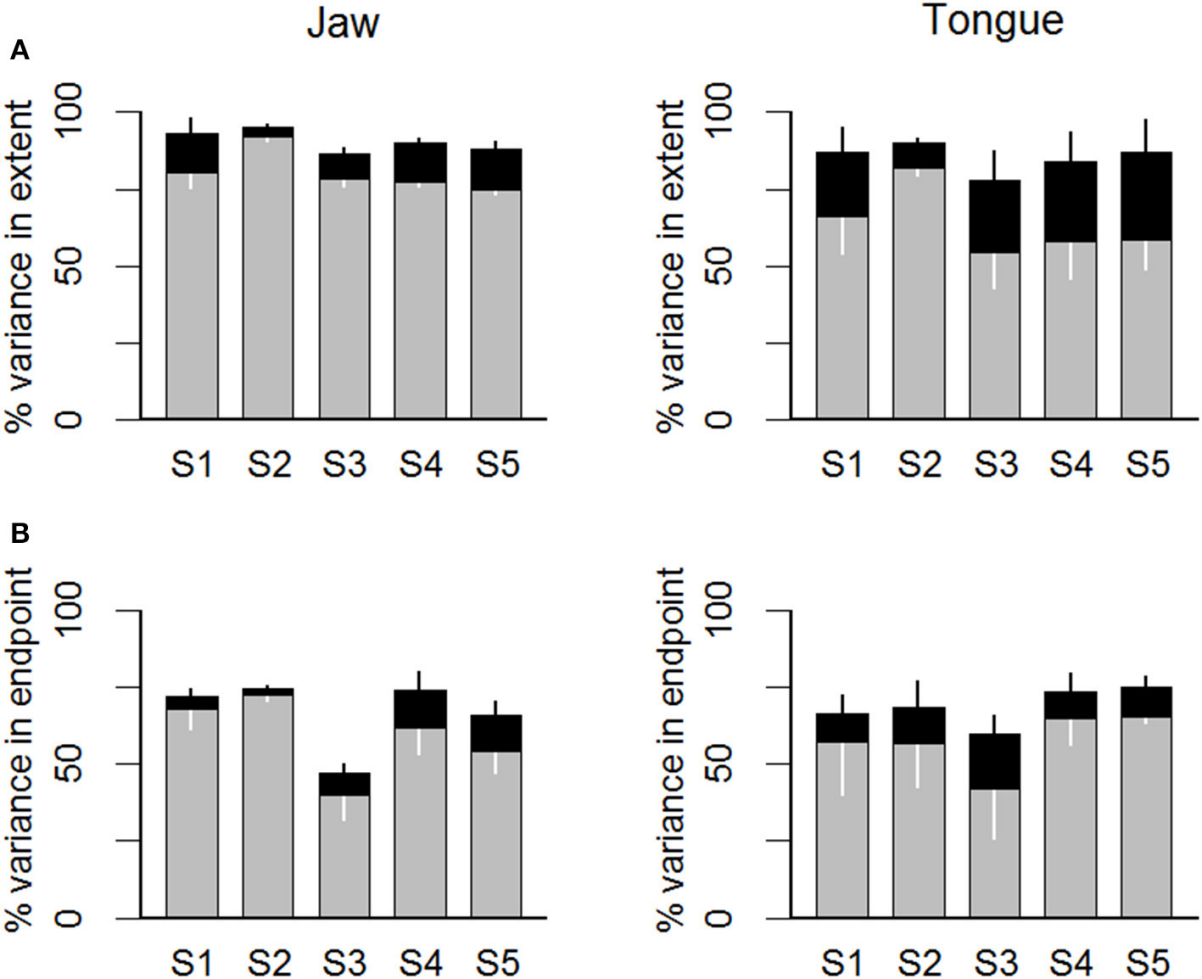
**Statistical estimates of feedback contributions for individual subjects (S1 … S5)**. The gray portion of each bar indicates the variance in movement extent **(A)** or in movement endpoint spatial coordinate relative to the major axis of the endpoint distribution ellipse **(B)** that was explained by the peak acceleration **(A)** or the articulator's major axis spatial coordinate at the time of peak acceleration **(B)**. The black portion of each bar indicates additional movement extent variance explained by adding movement duration (a variable assumed to be affected by feedback-based adjustments) in a multiple correlation model.

When considering the values of the kinematic parameters (upper row of panels in Figure 7) across subjects, the *total* variance in movement extent that was explained after adding movement duration as an additional predictor variable—together with peak acceleration—was in the range 87–95% for jaw movements and in the range 78–90% for tongue movements. Thus, the vast majority of variance in movement extent was accounted for with a simple multiple correlation model that predicted movement extent on the basis of the early kinematic parameter peak acceleration and feedback adjustments in movement duration. For the jaw, the *increase* in explained movement extent variance that resulted from taking movement duration into account ranged, across subjects and target syllables (i.e., 5 subjects × 2 syllable types × 2 consonants = 20 cases), from 3 to 13%. The *F* statistic calculated to test the increase in explained variance for each individual combination of subject and target syllable was statistically significant (uncorrected *p*-values) in 20/20 cases. For tongue movements, this *increase* in explained movement extent variance was approximately twice as large, ranging from 8 to 28%. The *F* statistic was again statistically significant in 20/20 cases.

Lastly, the same statistical model was also applied to the spatial coordinates data. Focusing here on the major axis data (i.e., the axis generally aligned with movement direction; data shown in the bottom row of panels in Figure 7), the *total* variance in movement endpoint that was accounted for when adding movement duration as a predictor variable—together with peak acceleration—was in the range 47–75% for jaw movements and 60–75% for tongue movements. In other words, the combination of the early peak acceleration spatial coordinates and movement duration explained most of the endpoint variance. For the jaw spatial coordinates, the *increase* in explained movement endpoint variance obtained by taking movement duration into account ranged, across subjects and target syllables, from 2 to 12%. The *F* statistic calculated to test the increase in explained variance for each individual combination of subject and syllable was statistically significant (uncorrected *p*-values) in 11/20 cases. For tongue spatial coordinates, the *increase* in explained movement endpoint variance was in the range 9–18%, and the calculated *F* statistic showed that the increase in explained movement endpoint variance was statistically significant in 15/20 cases.

## Discussion

We applied to speech movements a set of analyses previously used to assess the contributions of feedforward vs. feedback control mechanisms in limb and eye movements. For tongue and jaw movements during sentence-level speech, we measured traditional kinematic parameters (peak acceleration, peak velocity, movement extent) and the effectors' two-dimensional spatial coordinates at the time of those landmarks (at peak acceleration, peak velocity, and movement endpoint). Bivariate correlation coefficients (and the corresponding coefficients of determination) were used to examine the relationship between early kinematic variables (those related to peak acceleration and peak velocity) and overall movement variables (movement amplitude and the spatial coordinates at movement endpoint). A statistical technique based on multiple correlation (and the corresponding coefficient of multiple determination) was used to estimate the strength of feedback-based corrections.

Before summarizing the results and drawing conclusions based on the obtained correlations between early and final kinematics, it is important to acknowledge that low correlation coefficients, by themselves, would not necessarily indicate that the movements were mainly feedback driven and that relatively large adjustments took place after the time of peak acceleration and peak velocity. Hypothetically, a set of movements that all have the exact same start and end location and the same duration could have been planned with different time courses that cause the velocity profiles to have different shapes and different peak values. In this case, the correlations between peak acceleration or peak velocity and movement extent (or among the corresponding spatial coordinates) would be low even though the entire trajectory and time course may have been completed without any feedback-based adjustments. If, on the other hand, the correlations between early and final kinematics are high, then the data would, in fact, indicate that the total extent or endpoint location had already been determined by the time of peak acceleration or peak velocity (and, thus, that the movement was executed primarily under feedforward control). For this reason, the bivariate coefficients of determination can be interpreted as measures of how much feedforward mechanisms contributed *at a minimum* (the real contribution may be greater), but the unexplained variance cannot be interpreted as a measure of the relative strength of feedback contributions. Hence, the aforementioned method based on the coefficient of multiple determination was necessary to estimate the contribution of feedback-based mechanisms (Messier and Kalaska, 1999).

### Estimates of feedforward contributions

The obtained results suggest that articulatory speech movements are strongly feedforward driven. The group mean correlation coefficients between the values of kinematic parameters and movement extent were very high (*r* > 0.92 for peak velocity, *r* > 0.72 for peak acceleration) in all conditions (/t/ + V, /s/ + V, V + /t/, V + /s/) when jaw or tongue movements across trials with different vowel heights were considered. These correlation coefficients were always higher for jaw movements than for tongue movements—a finding that may be related to the more restricted degrees of freedom for jaw movements (rotation with minimal translation) as compared with tongue movements (hydrostatic deformation). Even when only movements to a single vowel target were considered, movement extent was still closely related to peak velocity (typically *r* = ~0.85) and peak acceleration (typically *r* ~ 0.60). Thus, even when all movements had the same target distance, small variations in the actually achieved distance were still reflected in the initial kinematics. These observations are consistent with the interpretation that orofacial speech movements are not extensively altered after the time at which peak acceleration is reached and definitely not after the time at which peak velocity is reached.

The correlation coefficients between spatial coordinates at the time of peak acceleration or peak velocity and movement endpoint point indicate that not only the overall movement extent but also the effector's location in the midsagittal plane (i.e., endpoint coordinates relative to the distribution of such coordinates across all trials) can be predicted reasonably well from earlier kinematic information. Given that group mean correlations based on the endpoint distributions' major axis typically were ~0.97 for the coordinates at peak velocity and ~0.76 for the coordinates at peak acceleration when different vowel targets were considered, it is appears that movement endpoints were indeed largely pre-determined. Again this was confirmed by the finding that even for within-vowel analyses, the coordinates at peak velocity and peak acceleration were strongly or moderately correlated, respectively, with the coordinates at movement endpoint.

Taken together, these findings based on the bivariate correlations suggest that the extent and endpoint location of tongue and jaw speech movements are almost completely predictable from kinematic information available at the time of peak velocity and that they are already largely determined at the time of peak acceleration. To put the obtained results in perspective by comparing speech and limb motor control, both jaw and tongue movements were associated with parameter-based or coordinates-based correlation coefficients that were higher than those reported for limb reaching movements (Messier and Kalaska, 1999), and this was especially the case for the more challenging situation in which all analyzed movements aimed for the same target distance. It should be noted that this apparent stronger weighting of feedforward control in speech production was observed despite the fact that these speech movements were performed with auditory feedback available, whereas the reaching movements in Messier and Kalaska (1999) were performed in the absence of visual feedback.

### Estimates of feedback contributions

Given that peak acceleration and peak velocity scaled strongly with movement extent, and thus appear to be determined prior to movement onset, we explored whether another variable may reflect online compensation for variability in the achieved values of peak acceleration and peak velocity. For this purpose, we tested how movement duration varied with peak acceleration and peak velocity when movements were planned to different targets (vowels of different height, thus with articulatory movements presumably planned to have different values of peak acceleration and velocity) or to a single target (a single vowel, thus with articulatory movements presumably planned to have the same values of peak acceleration and velocity) (Messier and Kalaska, 1999). These analyses showed that peak acceleration and movement duration were positively correlated across movements to different targets but more often negatively correlated across movements to a single target. Thus, the results did indeed raise the possibility that movement duration might be adjusted online to compensate for variability in peak acceleration. This hypothesis was then further tested with a multiple correlation model.

The reasoning behind the multiple correlation analysis was that (a) if another dependent variable correlates negatively with peak acceleration for movements planned to have the same extent, and (b) if adding that variable as an additional predictor causes a statistically significant increase in the proportion of explained movement extent variance as compared with that explained by the bivariate correlation between peak acceleration and movement extent alone, then it is likely that online adjustments in the added variable compensated for variability in the achieved peak acceleration. Here, we added movement duration as the additional predictor variable in the multiple correlation model, and the increase in explained movement extent variance after adding this variable to the model was considered an estimate of the strength of online adjustments.

Our results suggest that a speech movement's duration may indeed reflect, in part, feedback-driven compensation for variability in the achieved peak acceleration. For both jaw and tongue movements, adding movement duration to the model caused increases in explained variance for movement extent as well as for the spatial coordinates at movement endpoint. These duration-based estimates of feedback contributions were descriptively larger for tongue movements than for jaw movements, particularly in the case of movement extent. This finding complements the results discussed in the previous section, namely that separate estimates of feedforward contributions showed a stronger influence of planning for jaw movements than for tongue movements. Thus, the central nervous system (CNS) may adjust movement duration online to alter the originally planned movement and reach the desired movement target, and such online corrections may play a more substantial role in the control of multiple-degrees-of-freedom tongue movements vs. the primarily rotational jaw movements.

Unlike the situation for reaching movements (Messier and Kalaska, 1999), this data set based on speech movements suggests that compensation in duration was based only on peak acceleration rather than on both peak acceleration and peak velocity. We speculate that this different outcome may be related to the much shorter duration of articulatory speech movements vs. arm reaching movements. For example, Messier and Kalaska's (1999) illustration of individual movement trajectories shows durations in the range 500–1000 ms. Our own illustration of individual movement trajectories (Figure 1) shows durations in the range 120–150 ms. Hence, in the case of speech movements, only sensory information related to the initial acceleration may leave sufficient time for online adjustments to be implemented before the end of the movement. Given that peak velocity is only reached approximately halfway through the movement, sensory information obtained at this time during a speech movement (in our example trajectories in Figure 1, this was only 60–75 ms before the end of the movement) may be available too late to play a significant role in online compensatory adjustments. In general, however, kinesthetic sensory information about acceleration and velocity is available from muscle spindles that are present in jaw closing muscles (Kubota and Masegi, 1977) and in both intrinsic and extrinsic tongue muscles (Cooper, 1953; Walker and Rajagopal, 1959; Sussman, 1972; Kubota et al., 1975), possibly from additional sensory nerve endings in the tongue (Cooper, 1953; Adatia and Gehring, 1971), and from cutaneous mechanoreceptors in the facial skin (Ito et al., 2009).

Another interesting new finding of the present work is that the estimated strength of feedback contributions differs across the individual subjects. A first potential implication of this finding is that the analyses described here may be useful for quantifying inter-individual differences in the weighting of feedforward vs. feedback control mechanisms during speech production. Thus, these analyses may provide a novel approach for testing the hypothesis that individuals with certain speech motor disorders (for example stuttering, see Max et al., 2004) differ from typical speakers in terms of the underlying sensorimotor control strategies. A second potential implication of the observed variability across subjects is that different speakers may implement feedback based adjustments in different kinematic parameters. In the present study, we only tested duration as a dependent variable that could be adjusted during movement execution, but it may be fruitful for future studies to explore the possibility of parallel adjustments in additional movement parameters.

## Conclusion

Findings from this kinematic study of unperturbed articulatory movements indicate that jaw and tongue movements are mostly under feedforward control, but that there are substantial adjustments in movement duration that compensate for variability in the movement's initial acceleration. Descriptively, estimated feedforward contributions were greater, and estimated feedback contributions were smaller, for jaw movements than for tongue movements. Data for CV and VC syllables were highly similar, thus suggesting that jaw and tongue movements are controlled in generally similar ways for oral opening and closing gestures.

Of course, this work was associated with a number of limitations that should be addressed in future studies. First, given the exploratory nature of the study (examining different syllable types, consonants, and articulators) the number of calculated correlation coefficients and *F* statistics was very large, and the results presented here are based on uncorrected significance tests. Second, the included analyses made it necessary to exclude individual trials with more than one peak in the tangential velocity profile. Approximately 10% of the original data were excluded for this reason, and the effect of removing these trials is unknown. Third, our analyses did not take into account any potential influence of the orofacial system's biomechanics. We acknowledge that even within a single movement the role of biomechanical factors does not remain constant (Fuchs et al., 2011). Nevertheless, we believe that it is unlikely that factors such as muscle properties and interaction torques, rather than central planning and correction processes, can explain the overall results of this study. Not only are the analyses based on within-subject sets of movements to the same targets, there is also ample evidence suggesting that the CNS has access to detailed information about such factors and takes them into account during movement planning (Kawato and Wolpert, 1998; Flanagan and Lolley, 2001).

In sum, this study examined the contributions of feedforward and feedback mechanisms in the sensorimotor control of unperturbed speech movements by examining the relationship between initial and final kinematics. Specifically, we tested whether an articulator's movement extent or spatial coordinates at movement endpoint were accounted for by its peak acceleration, peak velocity, or spatial coordinates at those two kinematic landmarks. In addition, we tested whether the addition of movement duration to the model led to an increase in the proportion of variance in the final kinematics that was accounted for by variance in the early kinematics (after finding that movement duration correlated negatively with peak acceleration across movements toward the same target). We found that, similar to limb movements (Messier and Kalaska, 1999), articulatory movements of the jaw and the tongue are primarily under feedforward control but with important contributions from a feedback control system.

### Conflict of interest statement

The authors declare that the research was conducted in the absence of any commercial or financial relationships that could be construed as a potential conflict of interest.

## Footnotes

^1^Shifting the movement trajectories to a common starting point was implemented to avoid inflated correlations between early and late kinematics due to large variation in the actual starting points. For example, 10 straight-line vertical movements in the midsagittal plane with starting points that are always *x*+1 mm more posterior than the preceding trial would show a perfect correlation between the *x* coordinate at the time of peak acceleration and the *x* coordinate at movement endpoint. However, the predictability of these endpoint *x* coordinates based on the peak acceleration *x* coordinates is due entirely to the movements not originating from the same starting point. See also Figure 1.

^2^Following Messier and Kalaska (1999), we tested in the present work only movement duration as a potentially compensatory variable, but it is clear that future work should also explore whether other variables may add more explanatory power to the multiple correlation model. See also the Discussion section.

## References

[B1] Abbs J. H. Gracco V. L. (1984). Control of complex motor gestures: orofacial muscle responses to load perturbations of lip during speech. J. Neurophysiol. 51, 705–723. 6716120 10.1152/jn.1984.51.4.705

[B2] Abend W. Bizzi E. Morasso P. (1982). Human arm trajectory formation. Brain 105, 331–348. 10.1093/brain/105.2.331 7082993

[B3] Adatia A. K. Gehring E. N. (1971). Proprioceptive innervation of the tongue. J. Anat. 110, 215–220. 4259251 PMC1271091

[B4] Cooper S. (1953). Muscle spindles in the intrinsic muscles of the human tongue. J. Physiol. 122, 193–202. 13109753 10.1113/jphysiol.1953.sp004991 PMC1366190

[B5] Desmurget M. Grafton S. (2000). Forward modeling allows feedback control for fast reaching movements. Trends Cogn. Sci. 4, 423–431. 10.1016/S1364-6613(00)01537-0 11058820

[B6] Feng Y. Gracco V. L. Max L. (2011). Integration of auditory and somatosensory error signals in the neural control of speech movements. J. Neurophysiol. 106, 667–679. 10.1152/jn.00638.2010 21562187 PMC3154803

[B7] Flanagan J. R. Lolley S. (2001). The inertial anisotropy of the arm is accurately predicted during movement planning. J. Neurosci. 21, 1361–1369. 11160407 10.1523/JNEUROSCI.21-04-01361.2001 PMC6762220

[B8] Folkins J. W. Abbs J. H. (1975). Lip and jaw motor control during speech: responses to resistive loading of the jaw. J. Speech Hear. Res. 18, 207–219. 10.1044/jshr.1801.207 1127904

[B9] Folkins J. W. Abbs J. H. (1976). Additional observations on responses to resistive loading of the jaw. J. Speech Hear. Res. 19, 820–821. 10.1044/jshr.1904.820 1003959

[B10] Fuchs S. Perrier P. Geng C. Mooshammer C. (2006). What role does the palate play in speech motor control? Insights from tongue kinematics for German alveolar obstruents, in Speech Production: Models, Phonetic Processes, and Techniques, eds Harrington J. Tabain M. (New York, NY: Psychology Press), 149–164.

[B11] Fuchs S. Perrier P. Hartinger M. (2011). A critical evaluation of gestural stiffness estimations in speech production based on a linear second-order model. J. Speech Lang. Hear. Res. 54, 1067–1076. 10.1044/1092-4388(2010/10-0131) 21330648

[B12] Gordon J. Ghez C. (1987). Trajectory control in targeted force impulses. III. Compensatory adjustments for initial errors. Exp. Brain Res. 67, 253–269. 10.1007/BF00248547 3622688

[B13] Gracco V. L. Abbs J. H. (1985). Dynamic control of the perioral system during speech: kinematic analyses of autogenic and nonautogenic sensorimotor processes. J. Neurophysiol. 54, 418–432. 4031995 10.1152/jn.1985.54.2.418

[B14] Green J. R. Moore C. A. Higashikawa M. Steeve R. W. (2000). The physiologic development of speech motor control: lip and jaw coordination. J. Speech Lang. Hear. Res. 43, 239–255. 10.1044/jslhr.4301.239 10668666 PMC2890218

[B15] Green J. R. Moore C. A. Reilly K. J. (2002). The sequential development of jaw and lip control for speech. J. Speech Lang. Hear. Res. 45, 66–79. 10.1044/1092-4388(2002/005) 14748639 PMC2890215

[B16] Heath M. Westwood D. A. Binsted G. (2004). The control of memory-guided reaching movements in peripersonal space. Motor Control 8, 76–106. 14973339 10.1123/mcj.8.1.76

[B17] Ito T. Tiede M. Ostry D. J. (2009). Somatosensory function in speech perception. Proc. Natl. Acad. Sci. U.S.A. 106, 1245–1248. 10.1073/pnas.0810063106 19164569 PMC2633542

[B18] Kawato M. Wolpert D. (1998). Internal models for motor control. Novartis Found. Symp. 218, 291–304. discussion: 304–307. 9949827 10.1002/9780470515563.ch16

[B19] Kleinbaum D. G. Kleinbaum D. G. (1998). Applied Regression Analysis and Other Multivariable Methods. Pacific Grove, CA: Duxbury Press.

[B20] Kubota K. Masegi T. (1977). Muscle spindle supply to the human jaw muscle. J. Dent. Res. 56, 901–909. 10.1177/00220345770560081201 144744

[B21] Kubota K. Negishi T. Masegi T. (1975). Topological distribution of muscle spindles in the human tongue and its significance in proprioception. Bull. Tokyo Med. Dent. Univ. 22, 235–242. 132308

[B22] Kuehn D. P. Moll K. L. (1976). A cineradiographic study of VC and CV articulatory velocities. J. Phonet. 4, 302–320.

[B23] Löfqvist A. Gracco V. L. (2002). Control of oral closure in lingual stop consonant production. J. Acoust. Soc. Am. 111, 2811–2827. 10.1121/1.1473636 12083216 PMC2827774

[B24] Lucero J. C. Munhall K. G. Gracco V. L. Ramsay J. O. (1997). On the registration of time and the patterning of speech movements. J. Speech Lang. Hear. Res. 40, 1111–1117. 10.1044/jslhr.4005.1111 9328881

[B25] Max L. (2004). Stuttering and internal models for sensorimotor control: a theoretical perspective to generate testable hypotheses, in Speech Motor Control in Normal and Disordered Speech, eds Maassen B. Kent R. Peters H. F. M. van Lieshout P. Hulstijn W. (Oxford, UK: Oxford University Press), 357–388.

[B26] Max L. Guenther F. H. Gracco V. L. Ghosh S. S. Wallace M. E. (2004). Unstable or insufficiently activated internal models and feedback-biased motor control as sources of dysfluency: a theoretical model of stuttering. Contemp. Issues Commun. Sci. Disord. 31, 105–122.

[B27] Max L. Wallace M. E. Vincent I. (2003). Sensorimotor adaptation to auditory perturbations during speech: acoustic and kinematic experiments, in Proceedings of the 15th International Congress of Phonetic Sciences (Barcelona), 1053–1056.

[B28] Messier J. Kalaska J. F. (1999). Comparison of variability of initial kinematics and endpoints of reaching movements. Exp. Brain Res. 125, 139–152. 10.1007/s002210050669 10204767

[B29] Munhall K. G. Ostry D. J. Parush A. (1985). Characteristics of velocity profiles of speech movements. J. Exp. Psychol. Hum. Percept. Perform. 11, 457–474. 10.1037/0096-1523.11.4.457 3161986

[B30] Okadome T. Honda M. (2001). Generation of articulatory movements by using a kinematic triphone model. J. Acoust. Soc. Am. 110, 453–463. 10.1121/1.1377633 11508970

[B31] Ostry D. J. Keller E. Parush A. (1983). Similarities in the control of the speech articulators and the limbs: kinematics of tongue dorsum movement in speech. J. Exp. Psychol. Hum. Percept. Perform. 9, 622–636. 10.1037/0096-1523.9.4.622 6224895

[B32] Ostry D. J. Munhall K. G. (1985). Control of rate and duration of speech movements. J. Acoust. Soc. Am. 77, 640–648. 10.1121/1.391882 3882804

[B33] Perkell J. S. Zandipour M. (2002). Economy of effort in different speaking conditions. II. Kinematic performance spaces for cyclical and speech movements. J. Acoust. Soc. Am. 112, 1642–1651. 10.1121/1.1506368 12398469

[B34] Perrier P. Payan Y. Zandipour M. Perkell J. (2003). Influences of tongue biomechanics on speech movements during the production of velar stop consonants: a modeling study. J. Acoust. Soc. Am. 114, 1582–1599. 10.1121/1.1587737 14514212

[B35] Quinn L. Hamel V. Flanagan J. R. Kaminski T. Rubin A. (1997). Control of multijoint arm movements in Huntington's disease. Neurorehabil. Neural Repair 11, 47–60 10.1177/154596839701100108

[B36] Sokal R. R. Rohlf F. J. (1981). Biometry: The Principles and Practice of Statistics in Biological Research. San Francisco, CA: W. H. Freeman.

[B37] Sussman H. M. (1972). What the tongue tells the brain. Psychol. Bull. 77, 262–272. 10.1037/h0032374 4259645

[B38] Tremblay S. Shiller D. M. Ostry D. J. (2003). Somatosensory basis of speech production. Nature 423, 866–869. 10.1038/nature01710 12815431

[B39] Walker L. B. Rajagopal M. D. (1959). Neuromuscular spindles in the human tongue. Anat. Rec. 133, 438.

[B40] West G. L. Welsh T. N. Pratt J. (2009). Saccadic trajectories receive online correction: evidence for a feedback-based system of oculomotor control. J. Mot. Behav. 41, 117–127. 10.3200/JMBR.41.2.117-127 19201682

[B41] Wolpert D. M. Flanagan J. R. (2001). Motor prediction. Curr. Biol. 11, R729–R732 10.1016/S0960-9822(01)00432-8 11566114

[B42] Wolpert D. M. Flanagan J. R. (2010). Motor learning. Curr. Biol. 20, R467–R472. 10.1016/j.cub.2010.04.035 20541489

